# Origin of directionally tuned responses in lower limb muscles to unpredictable upper limb disturbances

**DOI:** 10.1371/journal.pone.0187006

**Published:** 2017-11-02

**Authors:** Ali Forghani, Theodore E. Milner

**Affiliations:** 1 University of Virginia Center for Applied Biomechanics, Charlottesville, Virginia, United States of America; 2 Department of Kinesiology and Physical Education, McGill University, Montreal, Quebec, Canada; Tokai University, JAPAN

## Abstract

Unpredictable forces which perturb balance are frequently applied to the body through interaction between the upper limb and the environment. Lower limb muscles respond rapidly to these postural disturbances in a highly specific manner. We have shown that the muscle activation patterns of lower limb muscles are organized in a direction specific manner which changes with lower limb stability. Ankle muscles change their activity within 80 ms of the onset of a force perturbation applied to the hand which is earlier than the onset of changes in ground reaction force, ankle angle or head motion. The latency of the response is sensitive to the perturbation direction. However, neither the latency nor the magnitude of the response is affected by stiffening the arm even though this alters the magnitude and timing of motion of the body segments. Based on the short latency, insensitivity of the change in ankle muscle activation to motion of the body segments but sensitivity to perturbation direction we reason that changes in ankle muscle activation are most likely triggered by sensory signals originating from cutaneous receptors in the hand. Furthermore, evidence that the latency of changes in ankle muscle activation depends on the number of perturbation directions suggests that the neural pathway is not confined to the spinal cord.

## Introduction

In many activities of daily living unpredictable forces are applied to the body through interaction of the upper limb with the environment. An unexpected tug on the arm, higher or lower resistance than anticipated when pulling or pushing or lifting a load or trying to open an unexpectedly sticky door are all actions which transmit forces to the body that disturb balance. When the upper limb is unexpectedly perturbed by an applied force the perturbation is transferred to other body segments and can disturb balance [1–4].

Unlike disturbances to balance originating from displacement of the support surface [5–9] force applied to the upper limb does not immediately produce a change in ground reaction force or ankle motion that could lead to sensory feedback from the leg. Yet there is evidence that ankle muscles can be activated as rapidly as elbow muscles in response to upper limb disturbances under conditions where the force direction is known [1]. Cordo and Nashner [1] ruled out vestibular sensory receptors and ankle muscle mechanoreceptors as the source of sensory information responsible for triggering the change in activation of ankle muscles based on the absence of head or ankle motion prior to the onset of changes in ankle muscle activation, However, their conclusions were based on video recordings that had relatively low temporal resolution (33 Hz) without any supporting statistical analysis. Furthermore, they did not speculate on which sensory inputs were responsible for triggering the earliest changes in ankle muscle activation. In their discussion they stated that “the degree of directional specificity provided by the motor command in not clear” and suggested that further experimentation was necessary.

We have recently conducted several studies [10–12] to advance our understanding of the postural responses described in [1]. These studies involved the same cohort of subjects as the current study. In [10], we characterized the pattern of change in muscle activation in relation to predictable and unpredictable perturbations to the arm in four orthogonal directions. We found that the muscle activity was tuned to the direction of the perturbation and that the response among muscles of the arm, trunk and leg were similarly organized whether the perturbation was presented in a predictable or unpredictable manner. We then varied stance width to test the hypothesis that the response to perturbations to the arm is context dependent, i.e. by altering balance in only the medial/lateral direction we showed that the amplitude of the response was altered only in lower limb muscles and only for the directions in which balance stability had been altered [11]. In that study, we also showed that the latency difference between the earliest changes in body kinematics and kinetics induced by the perturbation and the response in ankle muscles was not affected by stance width. By varying the amplitude of the perturbation we confirmed that the postural responses to arm perturbations scaled with the magnitude of the perturbation without altering their temporal organization [12]. From the results of these studies we determined that the rapid response in ankle muscles could not be triggered by signals from sensory receptors in leg muscles or cutaneous receptors in the foot because the ankle did not begin to move and ground reaction force did not begin to change until after the onset of ankle muscle activation. Furthermore, since the latency of trunk and head motion was similar to onset of ankle muscle activation it was unlikely that signals from sensory receptors of the vestibular system or trunk muscles could trigger the response. Therefore, we concluded that the sensory receptors responsible for triggering the response must be located in the upper limb.

In all of these previous studies, the elbow was flexed at the onset of the perturbation making the arm relatively compliant. To test whether sensory receptors associated with elbow or shoulder movement could be responsible for triggering the response in ankle muscles we altered the timing of movement of the elbow and shoulder by applying identical perturbations when the elbow was almost fully extended at the onset of the perturbation making the arm relatively stiff. In order to test whether the neural pathway might reside entirely within the spinal cord we compared the latency of the response with an identical protocol when there were only two perturbation directions. We reasoned that for a purely spinal pathway the latency should not be affected by the number of possible perturbation directions whereas a difference in latency would suggest that the pathway passed through higher centers such as the brainstem (cerebellum) or cerebral cortex.

## Methods

### Participants

Twelve (six male, six female) healthy subjects, without any known neurological, visual, or orthopedic disorders, were recruited from the McGill University student population to participate in the present study. The mean age of male subjects was 23.5 ± 3.3 years, mean height 1.76 ± 0.04 m and mean weight 66 ± 6 kg. The mean age of female subjects was 21.5 ± 4.1 years, mean height 1.67 ± 0.04 m and mean weight 57 ± 4 kg. All subjects self-identified as right hand dominant. All subjects provided written, informed consent prior to participation. Ethics approval for this study was received from the research ethics board of McGill University and all subjects provided written, informed consent prior to participation. The study was conducted according to the principles expressed in the Declaration of Helsinki.

### Protocol

**Fig 1:**
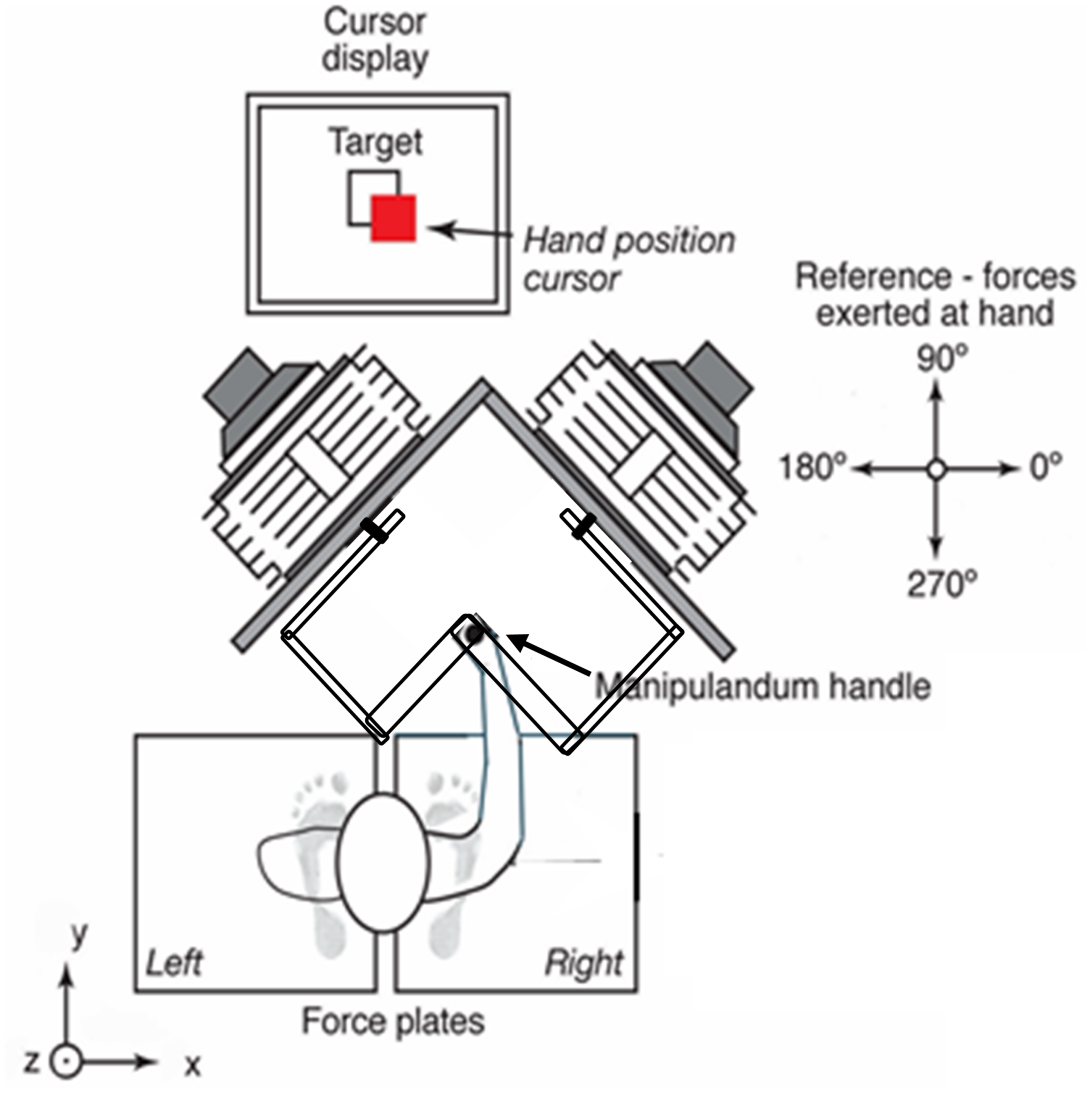
Experimental setup. Overhead view of apparatus, including motors, handle, target display and subject position. The coordinate reference frame is shown and force directions are indicated.

Subjects were required to hold a robotic joystick [13] in a vertical position while provided with visual feedback of the projection of the joystick position in the horizontal (*xy*) plane (Fig 1). Joystick position was displayed as a red square cursor (7×7 mm) on a vertically oriented 17″ LCD monitor, located approximately 1 m from the subject. The target was a white square (14×14 mm) located at the center of the monitor. The torque created by the weight of the joystick was compensated using a look-up table creating the sensation that the joystick was weightless. Each trial began by maintaining the cursor within the target zone for 1 s after which data acquisition began, with a force perturbation occurring at a random time between 0 and 0.5 s later. The perturbation profile consisted of a 150 ms force ramp, followed by a 3000 ms hold period and a 150 ms ramp-down to zero. Subjects were instructed to return the cursor into the target zone as rapidly as possible after the perturbation.

The subjects stood barefoot, grasping the joystick with their right hand. They grasped a plastic cylinder with bearing inserts that allowed it to slide along the metal shaft of the joystick. The cylinder prevented the subject from applying twisting torque to the joystick. The position of the cart and the height of the cylinder were adjusted so that the forearm was horizontal with the elbow flexed (condition F) at an angle of 120° (±5°) when the joystick was vertical. This was the arm configuration adopted for the majority of the test conditions. However, in one set of test conditions, subjects performed the task with the elbow extended (condition E). In this case the position of the cart was adjusted such that the cylinder remained at the same height but the elbow was fully extended when the joystick was vertical.

Stance width (the mediolateral distance between the medial and lateral calcanei) was set to 0.17 m, with 14° toe out (condition S) for comfortable normal stance [14]. A template placed on the force plates was used to standardize foot placement.

Prior to beginning the experiment, each participant exerted maximum isometric force three times against a fixed handle, in each of four directions: right, anterior, left and posterior (0°, 90°, 180°, and 270° relative to the positive *x*-axis). The mean maximal effort across subjects was 121.5± 29.9 N. The response to force amplitudes of 10%, 20% and 30% of the subject’s maximum force in the weakest direction were recorded. During pilot testing it was found that force amplitudes greater than 30% of a subject’s maximal effort led to fatigue and recovery maneuvers to avoid falling (e.g. stepping). Only the responses to the 30% force amplitudes were analyzed for the purpose of the present study.

Testing began with a familiarization period, during which subjects executed one trial in each direction and at each force level with the feet at normal stance width and the elbow flexed. To avoid fatigue, mandatory rest intervals were imposed after every nine trials. The duration of rest intervals was increased from 30 seconds up to 2 minutes as the experiment progressed to avoid the risk of cumulative fatigue effects. The subjects were also instructed that they could rest after a trial by simply moving the position cursor outside of the target square.

All subjects followed the protocol sequence listed in Table 1. Perturbation direction and amplitude were presented in a pseudo-random order. Subjects subsequently performed the task under several other conditions during the same session. The analysis and results of the subsequent conditions have been presented in previous studies [10–12] cited in the Introduction.

**Table 1:** Experimental protocol sequence.

Elbow Posture	Force Directions	Force Amplitudes	Number of trials (direction×force×trials)
F	90°, 270°	10%, 20%, 30%	2×3×9 = 54
E	90°, 270°	10%, 20%, 30%	2×3×9 = 54
F	0°, 90°, 180°, 270°	10%, 20%, 30%	4×3×9 = 108

F = elbow flexed, E = elbow extended

Only the 30% force amplitude condition was included in the present study since it produced the largest response and hence allowed latencies to be determined with the greatest accuracy. The rationale for comparing the response when the elbow was flexed and extended was to alter the compliance between the robot and the body. The extended elbow created a more rigid linkage between the robot and the body resulting in faster transmission of the disturbance to the body than when the elbow was flexed. We had two reasons for only applying perturbations in the anterior/posterior direction under this condition. First, the effect on the arm stiffness is much more dramatic in the anterior/posterior direction than in the left/right direction. Second, we wanted a condition where there were only two possible perturbation directions so that we could compare the latency of the change in muscle activation to the condition where there were four possible directions to investigate whether latency was linked to the number of possible responses.

### Data acquisition

#### Kinematic data

The position of the joystick was sampled at 1000 Hz and was used to represent the position of the subject’s right (perturbed) hand in the global coordinate system of the lab. The position of body segments was acquired at 200 Hz using a seven-camera MX3 motion-capture system (Vicon Peak). Thirty-nine 14mm diameter reflective spherical markers were placed according to the Plug-in-Gait model (Vicon Peak). Basic anthropometric measures required for the model (e.g. body mass and segment lengths) were made prior to acquiring data and used by the Plug-in-Gait model to compute joint angles and center of mass (COM) position.

#### Kinetic data

Hand force was acquired with a six-axis force transducer (ATI Mini-45) located at the base of the handle. Ground reaction forces and torques under each foot, in the *x*, *y*, and *z* directions were acquired from two tri-axial Bertec force plates (model FP4060). Force and torque were at sampled at 1000 Hz.

#### EMG

A DelSys Bagnoli 16-channel system was used to record surface EMG. Prior to electrode attachment, the skin was shaved and cleaned by rubbing with an alcohol pad to minimize electrical impedance of the electrode-skin interface. EMG was sampled at 1000 Hz. EMG was recorded bilaterally from the following muscles: tibialis anterior (TA), medial gastrocnemius (MG), peroneous longus (PL), tensor fascia latae (TFL), lumbar region of erector spinae (ES) and abdominal external oblique (AO). EMG was also recorded from the following right arm muscles: brachioradialis (BR), lateral head of triceps brachii (TC), anterior deltoid (AD) and posterior deltoid (PD). Electrodes were placed according to SENIAM guidelines [15].

### Preprocessing and data reduction

With the exception of onset detection, the kinematic and kinetic data were digitally filtered prior to analysis using a fourth-order, two-sided low-pass Butterworth filter with a cut-off frequency of 10 Hz.

Each trial was assessed to determine whether the subject maintained the joystick within the target zone for at least 1 s during the 3 s hold phase of the perturbation. Trials which did not meet this criterion were excluded from further analyses. Less than 1% of the trials were excluded (0.85%). Motion capture data obtained from three of the twelve subjects could not be used due to undetected detachment of some markers during the experiment. Therefore, the kinematic data (with the exception of hand motion which was acquired by the robot) were only analyzed for the other nine subjects (four female, five male).

Onsets of early changes in the EMG, kinematic and kinetic time series were determined automatically using the single-threshold detection algorithm proposed by Hodges and Bui [16]. Raw EMG signals were demeaned, full-wave rectified, and low-pass filtered at 50 Hz using a second-order Butterworth filter. The baseline EMG value was equal to the mean of a 500 ms window beginning 1 second prior to the perturbation onset. This baseline was then compared to the mean of a 50 ms moving window beginning 500 ms prior to the perturbation onset, moving forward one sample at a time until the mean of this window was found to exceed the baseline value by more than 2.5 standard deviations of the baseline. Once this condition was met, the algorithm stopped and the first sample of the moving window was used as an estimate for the onset time. A muscle was considered to be involved in the response if the activation onset occurred between within 300 ms of the perturbation onset. The same algorithm parameters were applied to estimate the onset of changes in ground reaction force. For determining onsets of the kinematic variables, the same algorithm was used but applied to their second derivative, i.e. the acceleration.

**Fig 2:**
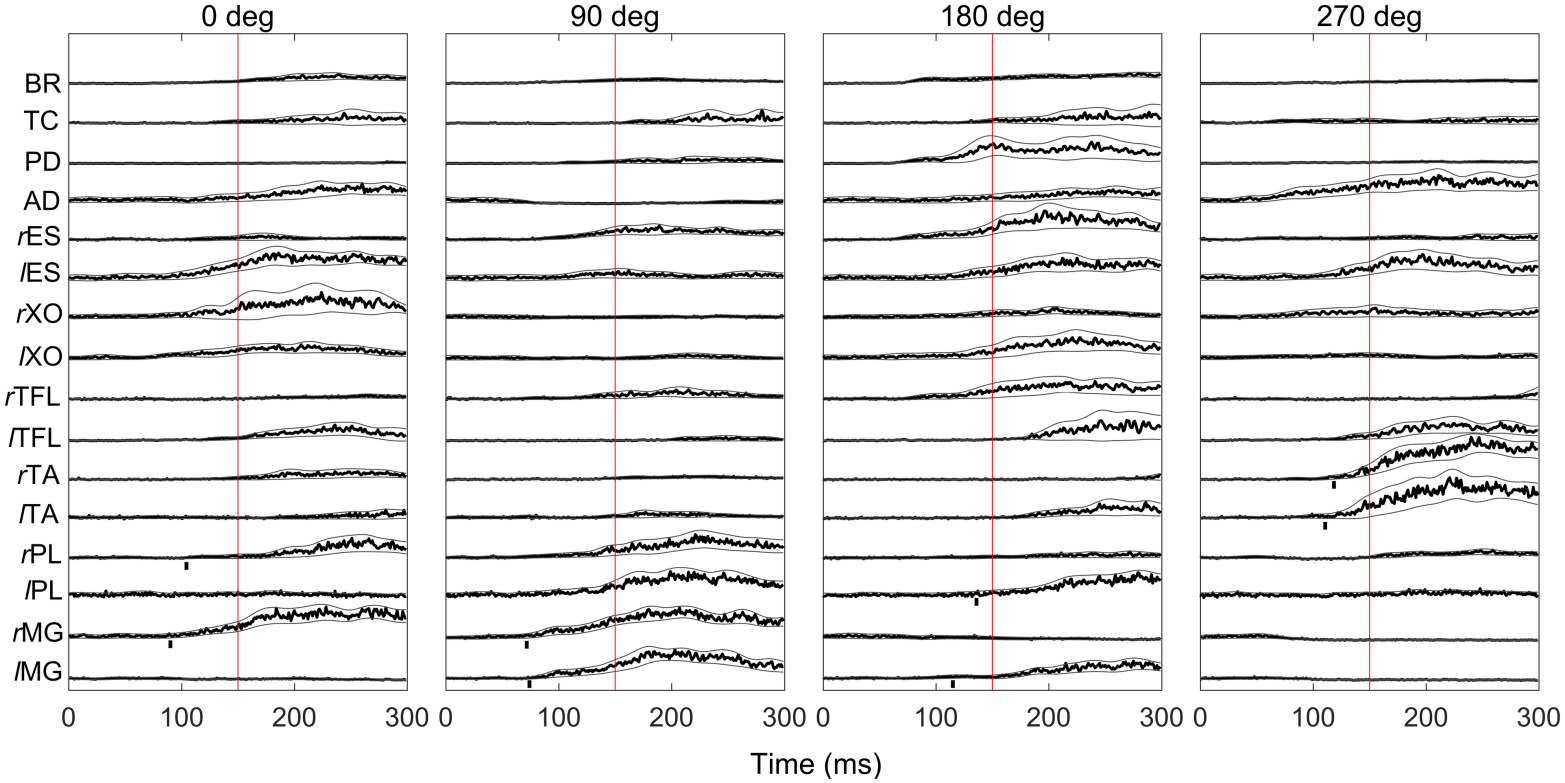
EMG response. Mean rectified, low-pass filtered EMG of all 16 muscles in response to perturbations in the four force directions. The traces represent the mean across subjects. The vertical lines delineate the interval between the perturbation onset and the end of the 150 ms interval over which the EMG was analyzed. Traces are shown from perturbation onset until 300 ms after the perturbation onset. BR, brachioradialis; TC, triceps brachii; PD, posterior deltoid; AD, anterior deltoid; *r*ES and *l*ES, right and left erector spinae; *r*AO and *l*AOl right and left abdominal external oblique; *r*TFL and *l*TFL, right and left tensor fascia latae; *r*TA and *l*TA, right and left tibialis anterior; *r*MG and *l*MG, right and left medial gastrocnemius; *r*PL and *l*PL, right and left peroneous longus.

Mean change in rectified EMG was analyzed for the 150 ms interval following perturbation onset. We selected this interval to include the range of responses known as automatic postural responses and to match the duration of the ramp phase of the perturbation. The mean rectified EMG over a 500 ms baseline interval 1000 to 500 ms prior to the perturbation was then subtracted. For each subject, the mean change in EMG for each muscle was normalized to the maximum response for that muscle across all trials in the [0, 150] ms time interval. A given ankle muscle responded to the perturbation with an increase in EMG for only a subset of the four force directions, as can been seen in Fig 2. Only those force directions for which a muscle consistently responded with an increase in activation were included in the analysis of onset latency and mean change in rectified EMG.

### Statistical analysis

The latencies and mean rectified EMG for each of the 9 trials of a given condition were averaged for each subject. Statistical analyses were performed on the resulting average values for the group of subjects. The effect of elbow angle or number of perturbation directions on the onset latency for each variable of interest was tested using repeated-measures, one-way ANOVA. For all analyses, the alpha level was set at 0.05.

## Results

### Patterned muscle response to perturbations

**Fig 3:**
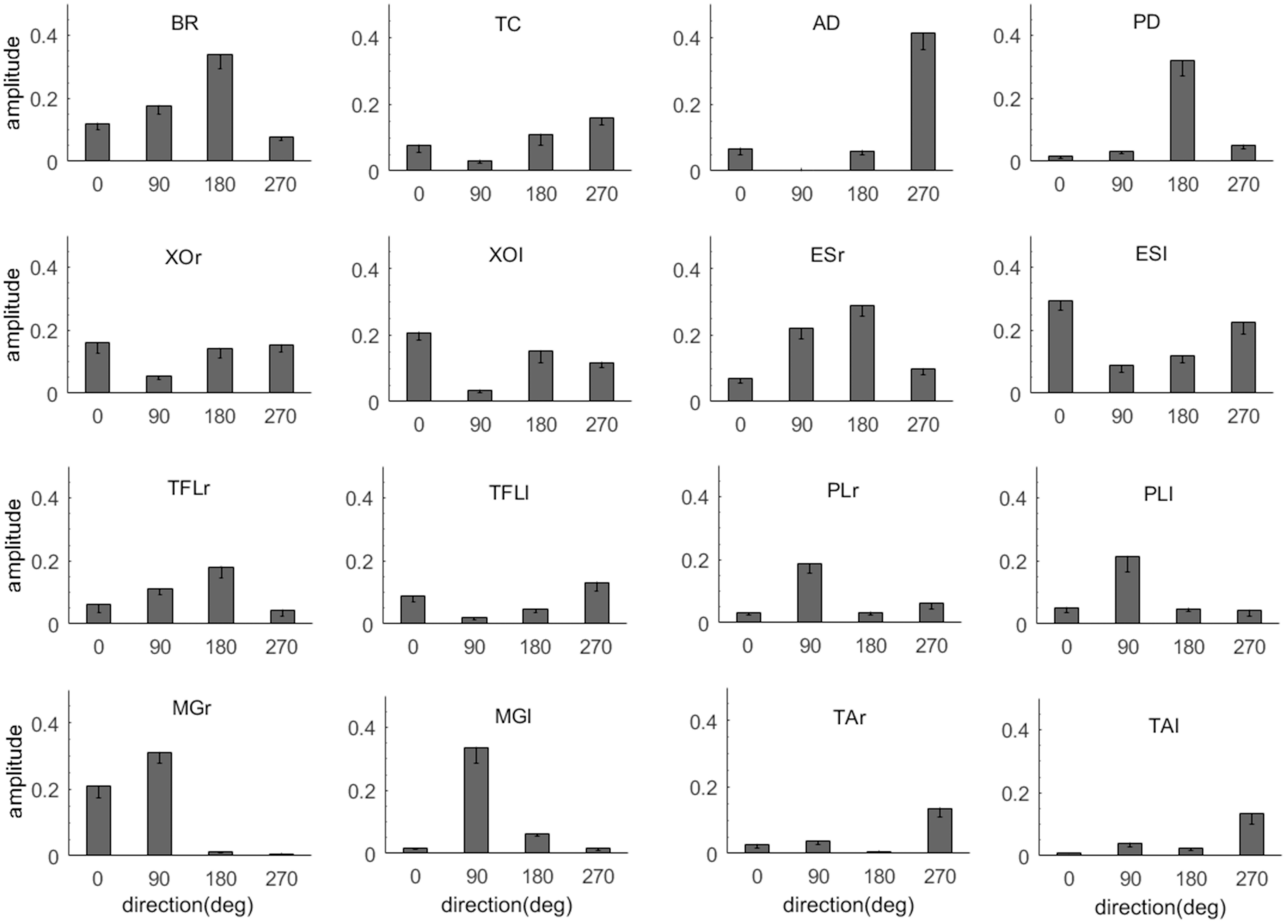
EMG response pattern. Mean change in rectified EMG for all 16 muscles across subjects during the [0, 150] ms time interval, calculated as a percentage of the maximum in that interval across all trials for each subject. The pattern of EMG change in lower limb muscles is unique for each force direction.

We have previously established that there was a distinct pattern to the change in activation of upper and lower limb muscles when a disturbance was applied to the hand [10]. This pattern was specific to the direction of the disturbance and is illustrated in Fig 2 where is can be seen that each muscle increases its activation for a specific subset of the four perturbation directions. The pattern does not appear to be organized in a symmetrical or reciprocal fashion which is not unexpected, particularly given that the force was not applied along the body midline, the limb and body mechanics are asymmetrical and the force direction rather than the displacement direction was controlled. The critical feature for the purpose of this study is that the ankle muscles respond preferentially for particular perturbation directions which establishes that the response is not simply an invariant triggered response but that information about force direction extracted from the sensory signal is used to control the response (Fig 3).

### Effect of elbow angle on perturbation response

Performing the task with the elbow extended had a significant effect on the kinematics of the arm in response to anterior/posterior perturbations compared to performing the task with the elbow flexed. The maximum hand displacement was larger for the flexed elbow than for the extended elbow in both anterior (41±18 mm compared to 32±17 mm) and posterior (43±27 compared to 34±24 mm) perturbations. A one-way repeated measures ANOVA showed that the effect of elbow angle was statistically significant (*F*(1,22) = 19, *p* = 0.001). When the elbow was extended it was not locked in hyperextension so the perturbation did produce some elbow movement albeit much less than when the elbow was flexed (< 0.5° compared to > 5°).

**Fig 4:**
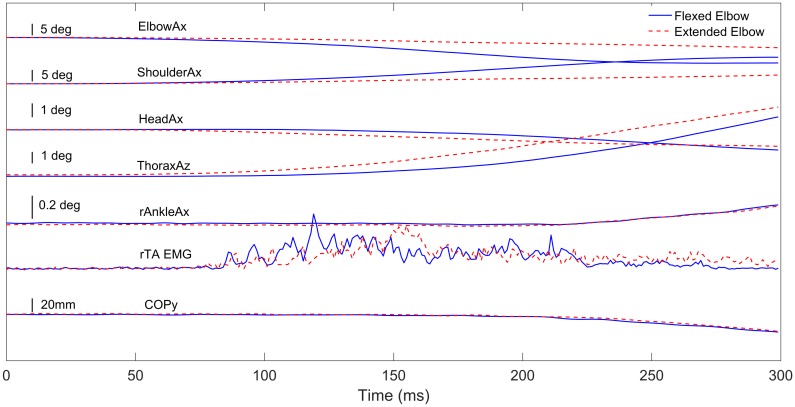
Kinematics, kinetics and EMG. Comparison of onsets of changes in kinematics, kinetics and tibialis anterior EMG of the right leg in response to hand perturbations in the posterior direction for flexed (solid blue lines) and extended (dashed red lines) elbow of a representative subject. Traces represent the mean of the subject for 9 trials. The perturbation begins at time 0. ElbowAx: change in elbow flexion/extension angle; ShoulderAx: change in shoulder flexion/extension angle; HeadAx: absolute anterior/posterior head rotation about the *x*-axis; ThoraxAz: absolute thorax rotation about the vertical axis; rAnkleAx: absolute anterior/posterior rotation of the right ankle joint about the *x*-axis; rTA EMG: rectified low-pass filtered EMG of the right tibialis anterior muscle; COPy: displacement of the right leg COP in the posterior (−*y*) direction.

We were interested in the timing of changes in mechanical variables relative to changes in the EMG of ankle muscles when the elbow was flexed compared to when it was extended. We first determined which mechanical variables changed before the EMG of ankle muscles and then examined the effect of elbow angle and stance width on these variables. Fig 4 compares the kinematic, kinetic and ankle EMG responses when the elbow was flexed and extended.

Table 2 lists the latencies of mechanical variables and EMG of ankle muscles relative to the onset of the perturbation for flexed and extended elbow conditions. The latency of the hand movement was derived from the robot encoder data whereas the latency of movement of the other body parts was derived from the motion capture data.

**Table 2:** Latencies (ms) for different elbow angles and perturbation directions.

	Flexed Anterior	Flexed Posterior	Extended Anterior	Extended Posterior
Hand	21±3	21±6	22±3	21±3
Wrist	37±6	34±4	*	*
Elbow	33±5	31±5	49±8	47±11
Shoulder	67±7	65±12	55±7	54±15
Thorax	66±12	56±9	52±18	55±16
Head	91±24	91±24	51±12	49±12
COM	70±21	67±24	71±24	71±27
*r*MG	81±8	^†^x	78±5	x
*r*TA	x	93±12	x	96±13
*r*PL	87±9	x	88±7	x
*r*GRF	132±24	140±21	129±18	145±18
*r*Ank	164±45	162±27	155±24	154±18

*Motion capture data were unreliable for wrist motion under the extended elbow condition

^†^x indicates that a muscle did not respond for that perturbation direction

Elbow angle had a significant effect on the latency of elbow movement both for both anterior (*F*(1,16) = 26, *p* = 0.0001) and posterior perturbations (*F*(1,16) = 16, *p* = 0.0011) and on the latency of head movement for both anterior (*F*(1,16) = 20, *p* = 0.0004) and posterior perturbations (*F*(1,16) = 22, *p* = 0.0002). There was also a significant effect on the latency of shoulder movement but only for anterior perturbations (*F*(1,16) = 13, *p* = 0.0022). Elbow angle had no significant effect on the onset latency of any of the other mechanical variables or the EMG of any of the ankle muscles.

We analyzed the effect of elbow angle on the change in EMG of ankle muscles in the [0,150] ms interval following the onset of the perturbation but found no effect of elbow angle on the change in EMG during this interval for any of the ankle muscles despite differences in hand displacement and onset latency of body segment movements between the flexed and extended elbow conditions.

### Effect of perturbation direction on response

The effect of perturbation direction (anterior/posterior and left/right) on the amplitude of the hand displacement was highly significant (*F*(1,16) = 187, *p*<0.0001). The hand displacement was approximately three times larger in the left/right direction (170±54 mm) than in the anterior/posterior direction (58±38 mm).

There was a significant effect of perturbation direction on EMG latency for some muscles. The onset of the change in EMG occurred later for rightward perturbations than for anterior perturbations for *r*MG (*F*(1,22) = 42, *p*<0.0001) and *r*PL (*F*(1,22) = 28, *p*<0.0001). The onset of the change in *l*MG and *l*PL EMG was not significantly affected by the perturbation direction. As noted previously, TA responded only to perturbations in the posterior direction so the effect of direction could not be tested for TA. The latencies are listed in Table 3 in the columns for four perturbation directions.

**Table 3:** Latency (ms) of ankle EMG.

	2 Directions Anterior/Posterior	4 Directions Anterior/Posterior	4 Directions Left/Right
*r*MG	79±9	85±9	113±12
*l*MG	80±11	90±13	94±13
*r*TA	90±16	120±13	^†^x
*l*TA	85±20	102±22	x
*r*PL	86±12	90±10	119±16
*l*PL	82±10	105±10	102±23

^†^x indicates that a muscle did not respond for that perturbation direction

Perturbations occurred in all four directions for the stance width condition whereas perturbations occurred in only two of the directions (anterior/posterior) for the elbow angle condition. We compared the onset latency of the response in ankle muscles for the data subset with flexed elbow and normal stance width under the conditions with two and four possible perturbation directions. This comprised the anterior direction for the MG and PL muscles and the posterior direction for the TA muscle, i.e. the directions in which these muscles responded to the perturbation. There was no effect of the number of perturbation directions on the latency of the *r*MG, *l*MG, *l*TA or *r*PL. However, there was an increase in the latency of *r*TA for posterior perturbations (*F*(1,22) = 25, *p*<0.0001) and *l*PL (*F*(1,22) = 32, *p*<0.0001) when there were four perturbation directions compared to two perturbation directions.

## Discussion

Disturbances to balance created by forces applied to the upper limb result in changes in activation of trunk and lower limb muscles which are specifically tuned to the direction of the disturbance. In the present study, we focused specifically on the earliest change in activation of ankle muscles. We found that ankle muscle EMG began to change before any detectable change in ground reaction force or ankle motion regardless of the direction of the perturbation. In some cases, the response began as early as 80 ms following the onset of the perturbation, although the latency depended on the direction of the perturbation. In the case of muscles of the right ankle, the change in EMG was delayed by about 30 ms for rightward perturbations compared to anterior perturbations. Increasing the stiffness of the arm in the anterior/posterior direction by extending the elbow caused the shoulder and head to begin moving earlier. However, under flexed and extended elbow conditions the response in ankle muscles occurred at the same latency despite differences in the magnitude and onset of the body segment motion. We had previously shown that stance width also had no effect on the latency [11]. Thus, we did not find evidence for a mechanical effect on the latency of the response in ankle muscles. On the other hand, the latency increased for certain muscles when the number of perturbation directions increased which would appear to be a cognitive rather than mechanical effect which makes it unlikely that the neural pathway responsible for the response is located entirely within the spinal cord.

Relatively fast changes in the EMG of postural muscles, particularly ankle muscles, in response to perturbations of the hand were investigated by [17]. They showed that small displacements of the thumb led to changes in EMG at latencies of 60–100 ms (depending on the location of the recording site) of muscles which could potentially be involved in stabilizing actions should balance be disturbed. However, their investigations were exploratory, limited to experiments conducted on only one or two subjects and involved predictable perturbations, i.e. perturbations were always in a single known direction. Furthermore, their experimental protocol involved manipulating conditions by trial and error until the desired responses were observed. They concluded that the sensory signals responsible for eliciting the change in EMG originated from muscle spindles in the thumb.

Cordo and Nashner [1] also demonstrated that the EMG of ankles muscles could change at very short latencies during self-initiated perturbations of the arm while pulling on a handle. Again, these perturbations were applied in a single predictable direction. Under one condition, perturbations were applied externally rather than being self-initiated. This condition was most analogous to the perturbations of the present study. However, the perturbation direction was known. Under this condition the onset of gastrocnemius muscle activation occurred 66 ms after perturbation onset. This was about 15 ms less than in our study and may be explained by knowledge of the perturbation direction in the earlier study and/or differences in the method used for determining the onset of muscle activation which was not described in the earlier study.

When interacting with the physical environment by means of upper limb actions the earliest source of information about the direction of an unexpected perturbation comes from cutaneous sensory receptors in the hand. This is followed by signals from muscle mechanoreceptors which are triggered by changes in muscle fiber motion or force. To control the action of lower limb muscles the central nervous system must transform the sensory signals into appropriate directional activation of lower limb muscles. Our results demonstrate that a transformation of sensory information can take place within approximately 80 ms of the onset of the perturbation. Lower limb motion and ground reaction force do not begin to change until after the onset of the change in muscle activation. This would rule out muscle mechanoreceptors and cutaneous mechanoreceptors in the leg as possible sources of the sensory trigger. The average latency of thorax acceleration onset was only 15 ms less than the average latency for the change in MG EMG. Only if transformation of the signal from sensory receptors in the thorax into commands to the ankle muscles occurred entirely within the spinal cord might there have been sufficient time for transmission of a motor command to ankle muscles, i.e. the neural pathway would have to be entirely confined to the spinal cord. The argument against a purely spinal pathway is the increase in latency for the change in *r*MG and *r*PL EMG under the condition of four perturbation directions compared to two perturbation directions. There is no logical reason why an increase in the number of perturbation directions should result in a 30 ms increase in the latency of the ankle muscle response if the neural pathway involved only spinal cord circuits.

More likely, the neural pathway involves either a reticulospinal [18–20] or a corticospinal circuit [21]. Although the existence of a spinal-brainstem-spinal pathway in humans has not been definitively established, Teng et al. [20] hypothesized that responses in ankle muscles to tapping the sternum or C7 spinous process were mediated by a reticulospinal pathway. The evidence for such a pathway is based on the response of reticulospinal neurons to stimulation of forelimb and hindlimb cutaneous nerves in the cat [18, 19]. Although there is no evidence that muscle mechanoreceptors project to reticulospinal neurons, we do not rule out the possibility. In [20] the latency between the tap and the onset of a change TA EMG was reported to be 50–55 ms. We consider first the possibility that mechanoreceptors (cutaneous or muscle receptors) responding to movement of the thorax triggered the response in ankle muscles. Our perturbation did not produce any detectable motion of the thorax until at least 55 ms after the onset of the perturbation. Assuming that the TA response is mediated by the same pathway as in [20], the latency to onset of change in TA EMG could not be less than 105 ms. Since this would exceed the observed latency by at least 10 ms (Table 2) we conclude that it is unlikely that the response in ankle muscles was triggered by sensory receptors in the thorax. The latency to onset of shoulder movement is similar to that of the thorax. Therefore, it is equally unlikely that mechanoreceptors around the shoulder would have triggered the response in ankle muscles. The elbow began to move earlier than the thorax or shoulder, i.e. between 31–33 ms after perturbation onset when the elbow was flexed. Given that the longer conduction distance should not add more than 5 ms to the latency between perturbation onset and change in TA EMG the predicted latency would be in the range of 86–93 ms which is comparable to what we observed with the elbow was flexed. However, when the elbow was extended elbow movement began about 16 ms later so the predicted latency to change in TA EMG would be 16 ms longer making it unlikely that mechanoreceptors around the elbow could consistently trigger the response in ankle muscles. With the elbow flexed wrist movement began at almost the same time as elbow movement. Since the conduction delay for mechanoreceptors around the wrist would add only a few milliseconds to the onset of change in TA EMG compared to the elbow it is possible that the response in ankle muscles was triggered by movement of the wrist, although we do not know whether this would have been the case when the elbow was extended due to unreliable data. Finally, we consider cutaneous receptors in the hand. Assuming that they are activated close to the same time as the onset of the perturbation, their signals would arrive at the brainstem before those of any other afferents of the upper limb. Given that conduction time to the brainstem for median nerve stimulation is about 14 ms [21] the predicted response latency between perturbation onset and change in TA EMG, based on the pathway proposed in [20] would be of the order of 70 ms. Thus, for a spinal-brainstem-spinal pathway an additional 20 ms of brainstem processing time would have to be interposed for unpredictable bidirectional perturbations to the hand compared to unidirectional body taps [20] in order for cutaneous receptors in the hand to be considered as viable candidates for triggering the response in ankle muscles.

We now consider the possibility that the pathway is transcortical. The latency would be determined by the sum of conduction time from the sensory receptors to the cortex, cortical processing time and conduction time from the cortex to the ankle muscles. The conduction time from the primary motor cortex to ankle muscles is about 30 ms as established from transcortical stimulation [22]. Given that the latency to change in MG EMG was 80 ms activation of the primary motor cortex would have to occur no later than 50 ms following perturbation onset. The latency from stimulation of the median nerve to change in EEG over the somatosensory cortex is about 20 ms whereas the latency from imposed movement of the wrist to change in EEG over the somatosensory cortex is about 25 ms. This is followed by change in EEG over the primary motor cortex about 10 ms later [21]. Assuming that cutaneous receptors in the hand are activated at perturbation onset, they could activate the motor cortex within 35 ms. However, given that motion of the wrist does not begin until about 35 ms after perturbation onset sensory signals from mechanoreceptors around the wrist could not activate the motor cortex sooner than 70 ms after perturbation onset. The latency is also problematic for mechanoreceptors associated with elbow movement. Even if we assume that they are activated 5 ms before those associated with wrist movement (Table 2) and that the conduction time to the cortex is 5 ms less the latency to activate the motor cortex would still be 60 ms. Since the shoulder and thorax did not begin to move until more than 50 ms following perturbation onset their associated mechanoreceptors can be eliminated from consideration. In support of cutaneous receptors in the hand triggering an ankle muscle response Misiaszek et al. [23] showed that rapid forward displacement of a lightly touched object produced a change in activation of the TA muscle with a latency of approximately 80 ms consistent with a stabilizing response to backward tilt of the body. The sensory trigger more likely originated from cutaneous receptors than muscle mechanoreceptors given that the object slid under the fingers rather than pulling on the hand. Lowrey et al. [24] conducted a study that was analogous to our study where the perturbation was a step force applied during movement to a target. They observed responses in ankle muscles (MG and TA) at comparable latencies to those of our study. Their study focused primarily on whether responses in upper limb muscles preceded those of lower limb muscles. They did not record the motion of the arm or speculate on which sensory receptors were responsible for triggering the response in lower limb muscles but did comment on the possibility of a rapid transcortical pathway between the upper and lower limbs.

Based on analysis of the latency between presumed onset of receptor activation and observed onset of change in ankle muscle EMG we propose that cutaneous receptors in the hand are a viable trigger for the response in ankle muscles whether the neural pathway is a spinal-brainstem-spinal pathway or a transcortical pathway. It is possible that mechanoreceptors around the wrist or elbow could trigger the ankle muscle response for a spinal-brainstem-spinal pathway under certain conditions but not for a transcortical pathway. Additional studies are currently underway to obtain more direct evidence for the role of cutaneous receptors in the hand when perturbations are applied to a handheld object.
